# Ionizing Radiation induction of cholesterol biosynthesis in Lung tissue

**DOI:** 10.1038/s41598-019-48972-x

**Published:** 2019-08-29

**Authors:** Erica Werner, Andrew Alter, Qiudong Deng, Eric B. Dammer, Ya Wang, David S. Yu, Duc M. Duong, Nicholas T. Seyfried, Paul W. Doetsch

**Affiliations:** 1 Department of Radiation Oncology, Winship Cancer Institute, Emory University School of Medicine, Atlanta, Georgia United States of America; 2 Department of Biochemistry, Emory University School of Medicine, Atlanta, Georgia United States of America; 3 Emory Proteomics Core, Emory University School of Medicine, Atlanta, Georgia United States of America; 4 Laboratory of Genomic Integrity and Structural Biology, NIH, National Institute of Environmental Health Sciences, Research Triangle Park, North Carolina, United States of America

**Keywords:** Cancer, Radiotherapy

## Abstract

While evidence supporting the notion that exposures to heavy ion radiation increase the risk for cancer and other disease development is accumulating, the underlying biological mechanisms remain poorly understood. To identify novel phenotypes that persist over time that may be related to increased disease development risk, we performed a quantitative global proteome analysis of immortalized human bronchial epithelial cells (HBEC3-KT) at day 7 post exposure to 0.5 Gy Fe ion (600 MeV/nucleon, Linear Energy Transfer (LET) = 175 keV/μm). The analysis revealed a significant increase in the expression of 4 enzymes of the cholesterol biosynthesis pathway. Elevated expression of enzymes of the cholesterol pathway was associated with increased cholesterol levels in irradiated cells and in lung tissue measured by a biochemical method and by filipin staining of cell-bound cholesterol. While a 1 Gy dose of Fe ion was sufficient to induce a robust response, a dose of 5 Gy X-rays was necessary to induce a similar cholesterol accumulation in HBEC3-KT cells. Radiation-increased cholesterol levels were reduced by treatment with inhibitors affecting the activity of enzymes in the biosynthesis pathway. To examine the implications of this finding for radiotherapy exposures, we screened a panel of lung cancer cell lines for cholesterol levels following exposure to X-rays. We identified a subset of cell lines that increased cholesterol levels in response to 5 Gy X-rays. Survival studies revealed that statin treatment is radioprotective, suggesting that cholesterol increases are associated with cytotoxicity. In summary, our findings uncovered a novel radiation-induced response, which may modify radiation treatment outcomes and contribute to risk for radiation–induced cardiovascular disease and carcinogenesis.

## Introduction

Understanding the scope of biological responses to radiation is of increasing importance as exposures to radiation of human populations are rising due to medical diagnostics and treatment, potential radio-nuclear accidents, and future space travel^1^.

The most significant known effect of low to moderate doses of radiation, which cause DNA damage that is readily repairable in normal cells, is an increased risk of cancer^2^. While the underlying mechanisms remain largely unknown, current models for radiation-induced carcinogenesis propose that radiation increases the probability of initiating mutations as well as the sequential accumulation of driver mutations in susceptible cell populations^3^. However, cancer as a disease results from a process influenced by multiple factors such as environmental and genetic constitution, and therefore could be affected by additional cellular responses elicited by radiation not related to mutated DNA^4^. Furthermore, non-DNA damage-related radiation responses might also play a role in the emergence of non-cancer pathological outcomes, including cardiovascular disease, fibrosis, cataracts and central nervous system effects.

Initial DNA damage caused by radiation triggers mechanisms to restore genetic stability and cellular homeostasis. We and others have previously shown that a single exposure to a moderate dose of radiation in the range of 1 Gy, which causes DNA damage that is readily repaired by normal cells, can induce cellular stress responses that persist for weeks^5^. Such stress response associated with DNA damage repair processes could influence later outcomes by affecting the establishment and permanency of chromosomal aberrations and DNA mutations, as well as modulating cell fate and function in the exposed cell population. Persistent responses to radiation may provide molecular targets to mitigate the emergence of late deleterious effects as well as sources for potential biomarkers to monitor responses to radiation at the individual level and across an extended period of time.

We analyzed the effects of exposure to high mass and energy (HZE) particle radiation, because of its overall higher biological effectiveness and carcinogenic potential^6^. Furthermore HZE particle radiation is a ground-based model for space radiation and relevant for particle beam therapy of human cancers. To gain biological knowledge about the scope of the cellular processes altered following exposure to radiation, we analyzed quantitative changes in the proteome, which captures the effects of changes in gene expression, translation and post-translational regulation and is a close reflection of cellular phenotype. We employed the human bronchial epithelial cell line HBEC3-KT, a non-tumorigenic immortalized cell line as an *in vitro* model for lung epithelia, which is a radiosensitive organ susceptible to radiation-induced cancer and late toxicity.

## Results

**Figure 1:**
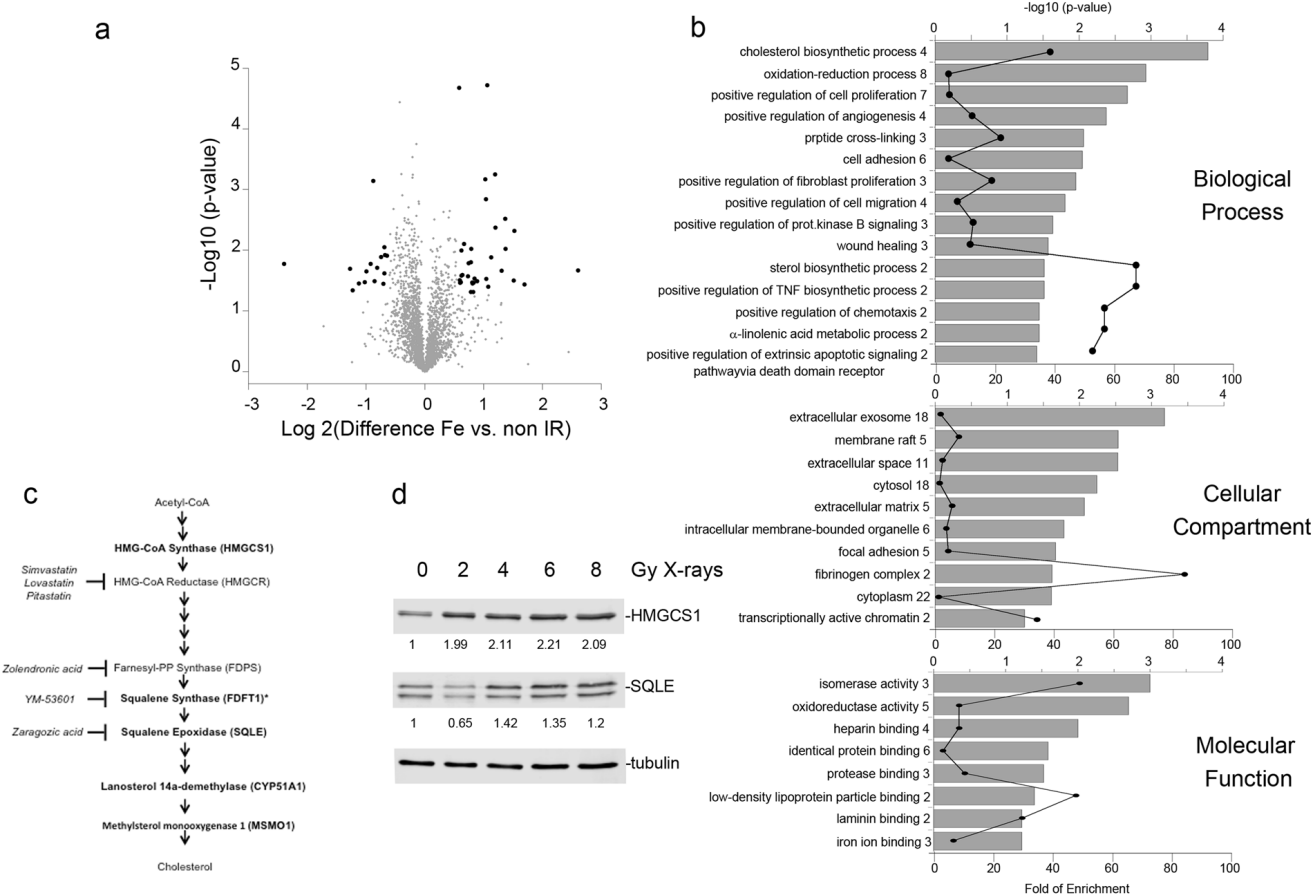
Quantitative global proteomic analysis of the cellular response at day 7 following a 0.5 Gy Fe ion exposure. (**a**) Volcano plot displaying the distribution of the proteins identified in all samples and proteins differentially regulated significantly by particle radiation exposure highlighted in bold. (**b**) Top GO terms identified for the list of differentially expressed proteins following annotation analysis in DAVID. The graphs display the significance (grey bar) and the relative enrichment (line graph) of proteins in the list compared to a random sample. Next to the GO term, the number indicates the number of proteins in the list included in the category. (**c**) Five of the significantly induced proteins (gene symbol in parenthesis) belong to the cholesterol biosynthetic pathway and are highlighted in bold. * = FDFT1 was induced two-fold, but did not pass the FDR filter setting of our analysis. The diagram includes the inhibitors employed in the experiments. (**d**) Western blot analysis for the expression of HMGCS1 and SQLE in 100 μg protein extracts prepared form HBEK3-KT at day 7 post exposure to the indicated X-rays dose. The numbers indicate fold change from non-irradiated samples after correction for loading.

We exposed HBEC3-KT to moderate radiation doses ranging between 0.5 to 1 Gy of Fe ion and 2 to 8 Gy X-rays, doses within a therapeutic range and known to increase cancer risk in a normal human population^7^. We have previously shown that at day 7, cells that have been exposed to 1 Gy of low or high LET radiation are actively proliferating within the context of numerous altered cellular processes such as oxidative stress, genomic instability and pro-inflammatory cytokine production^5,8,9^. To discover novel relevant cellular phenotypes that are persistently affected, we conducted a label-free global proteome analysis of cells at day 7 post-exposure to 0.5 Gy Fe ion. A dose of 0.5 Gy was previously shown to cause detectable cytogenetic damage in lung cells obtained from irradiated mice^10^. Analysis of triplicate samples revealed that among 2706 proteins identified and quantified in all 6 samples, radiation exposure changed the expression of 51 proteins at a statistically significant level (Supplementary Table 1), as visualized in a volcano plot (Fig. 1a). Among the top three proteins induced by Fe ion exposure is IL-1α, which we have previously identified by ELISA as a radiation-induced cytokine driving the production of IL-8 and other inflammatory molecules^8^. Thus, the current approach detects some of the molecules we have previously identified by biochemical methods. Other proteins induced are Fatty Acid Desaturase 1 and 2 (Supplementary Table 1), enzymes that regulate the synthesis of polyunsaturated fatty acids and therefore indirectly control the availability of precursor molecules for the pro-inflammatory mediators arachidonic acid, eicosanoids and prostaglandins^11,12^, pointing to a broad lipogenic and inflammatory phenotype that comprises cytokines and lipid metabolites.

The significantly altered proteins were functionally annotated and mapped to biological processes employing the bioinformatics DAVID annotation tool. The analysis revealed a significant increase of proteins involved in tissue repair and remodeling such as molecules promoting cell proliferation, angiogenesis, wound healing, chemotaxis, and the cellular interaction with the extracellular matrix (Fig. 1b). Most interestingly, the analysis revealed 4 enzymes involved in the *de novo* cholesterol biosynthesis pathway (Fig. 1c). We validated the mass spectrometry findings by western blot analysis of the expression of 2 of the enzymes identified in the proteome, HMGCS1 and SQLE in HBEC3-KT exposed to increasing X-rays doses of 2, 4, 6 and 8 Gy. As seen in Fig. 1d, low LET radiation increased the relative expression of the enzymes, with a threshold of 4 Gy, without further increase at higher dose. These results indicate that low and high LET radiation induce the expression of multiple enzymes in the cholesterol biosynthesis pathway.

**Figure 2:**
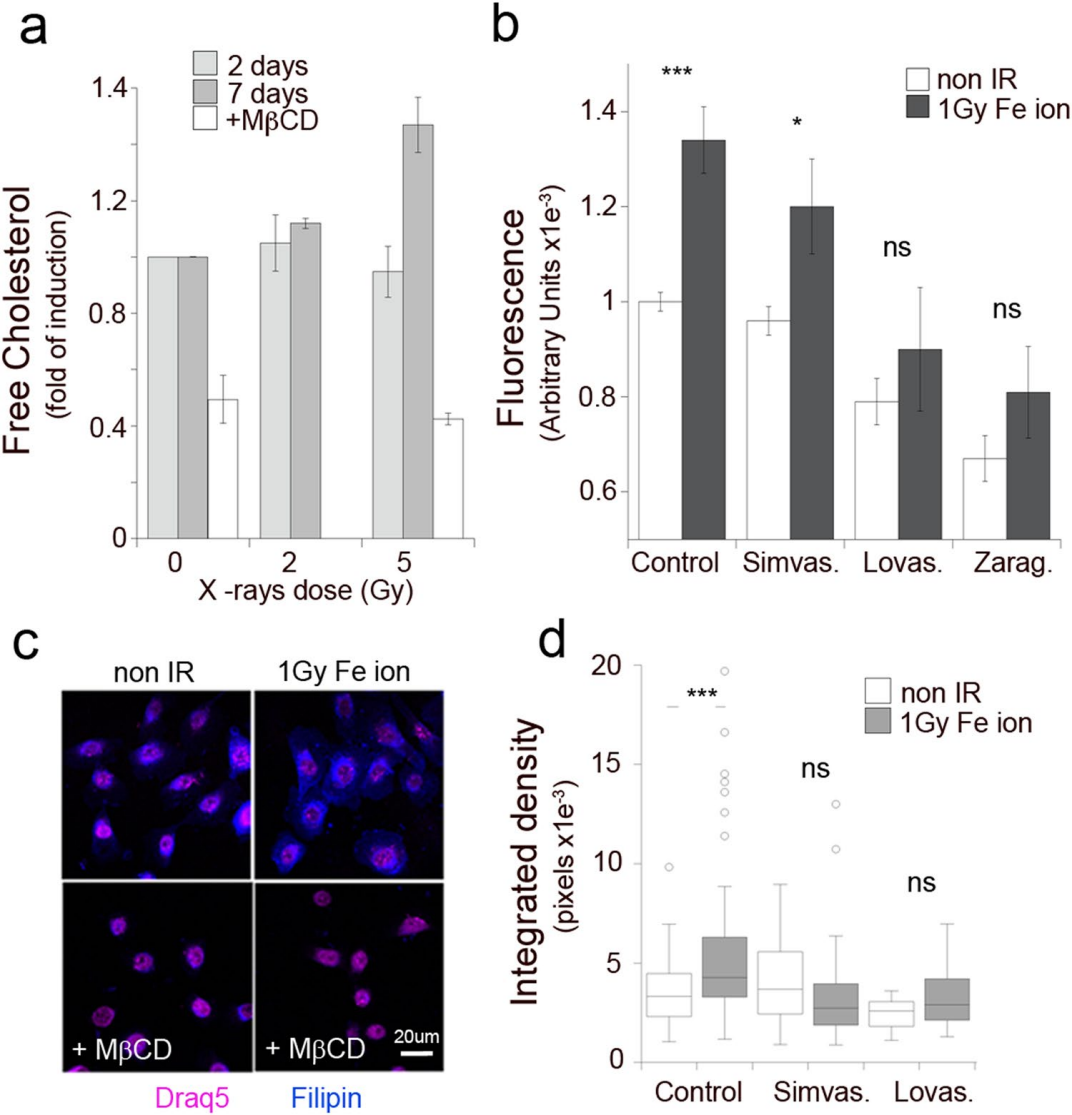
Exposure to Fe ion radiation increases cell-associated cholesterol. (**a**) Free cholesterol measured with the Amplex Red assay in HBEC3-KT cells at day 2 (light gray) or 7 (dark gray) following exposure to 2 or 5 Gy X-rays. White bars represent samples that were treated with 10 mg/ml Methyl-beta-cyclodextrin for 1 h prior to analysis. Error bars represent standard error of 2 replicate experiments. (**b**) Free cholesterol measured with the Amplex Red assay in HBEC3-KT cells at day 7 following mock irradiation (white bars) or exposure to 1 Gy Fe ion (gray bars). Some samples were incubated with vehicle (Control), 10 μM Simvastatin, 10 μM Lovastatin or 40 μM Zaragozic acid for 18 h prior to analysis. Error bars represent standard error of 3 replicates. T-test *p < 0.05, ***p < 0.005. (**c**) Filipin fluorescence microscopy at day 7 post–irradiation with 1 Gy Fe ion of HBEC3-KT cells pre-treated or not for 1 h with 10 mg/ml Methyl-beta-cyclodextrin before fixation. Draq5 is a cell permeable DNA binding fluorescent probe with far-red emission employed as a counterstain. (**d**) Quantification of cell-associated filipin fluorescence of HBEC3-KT cells at day 7 post-exposure to 1 Gy Fe ion. Some cultures were treated for 18 h prior staining with vehicle and 10 μM Simvastatin or Lovastatin. Box plots indicate the mean value and standard deviation of measurements in 35 or more cells per condition. Paired T-test, ****p < 0.001.

Cellular levels of cholesterol are tightly regulated to balance availability of essential metabolites for cellular function with toxicity. Therefore, to test whether the observed changes in enzyme levels are sufficient to affect cellular cholesterol levels, we measured total cholesterol content in HBEC3-KT following exposure to radiation employing two methods: an assay based on the enzymatic oxidation of cholesterol coupled to Amplex Red fluorescence and another based on filipin III from *Streptomyces filipenses*, a polyene antifungal antibiotic with a chromophore that undergoes a shift in fluorescence when bound to non-esterified cholesterol^13^. Both assays detect cell-associated cholesterol levels, which were proportional to cell number (not shown) and sensitive to treatment with inhibitors of cholesterol synthesis (Fig. 2b,d) or chelation with methyl-beta-cyclodextrin (Fig. 2a,c). We detected increased levels of cholesterol in HBEC3-KT cells at day 7-post exposure to 5 Gy X-rays, a dose above the threshold where enzymes are detected by western blot, but not at an earlier time point or at a lower dose of 2 Gy (Fig. 2a). An increase in cholesterol levels was readily detected at day 7 following 1 Gy Fe ion exposure (Fig. 2b). Thus, based on the measurements in panels A and B, we estimate a relative biological effectiveness (RBE) of 5 for the response induced by particle radiation compared to X-rays. Basal and radiation-induced cholesterol levels were reduced in cells treated with inhibitors targeting multiple enzymes in the cholesterol biosynthetic pathway when added 18 h prior to the measurements (Fig. 2b,d). While different inhibitors showed variable efficiency, all reduced the basal cholesterol content in non-irradiated cells and the increase induced by radiation. An increase in cell-associated cholesterol induced by exposure to Fe ion and a reduction in cells treated with statins was also evident with filipin staining, which showed increased labeling of the plasma membrane and intracellular compartments (Fig. 2c). Thus, these results indicate that *de novo* synthesis contributes to the total pool of cholesterol in HBEC3-KT cells and that exposure to low and high LET radiation increases the biosynthesis of cholesterol.

**Figure 3:**
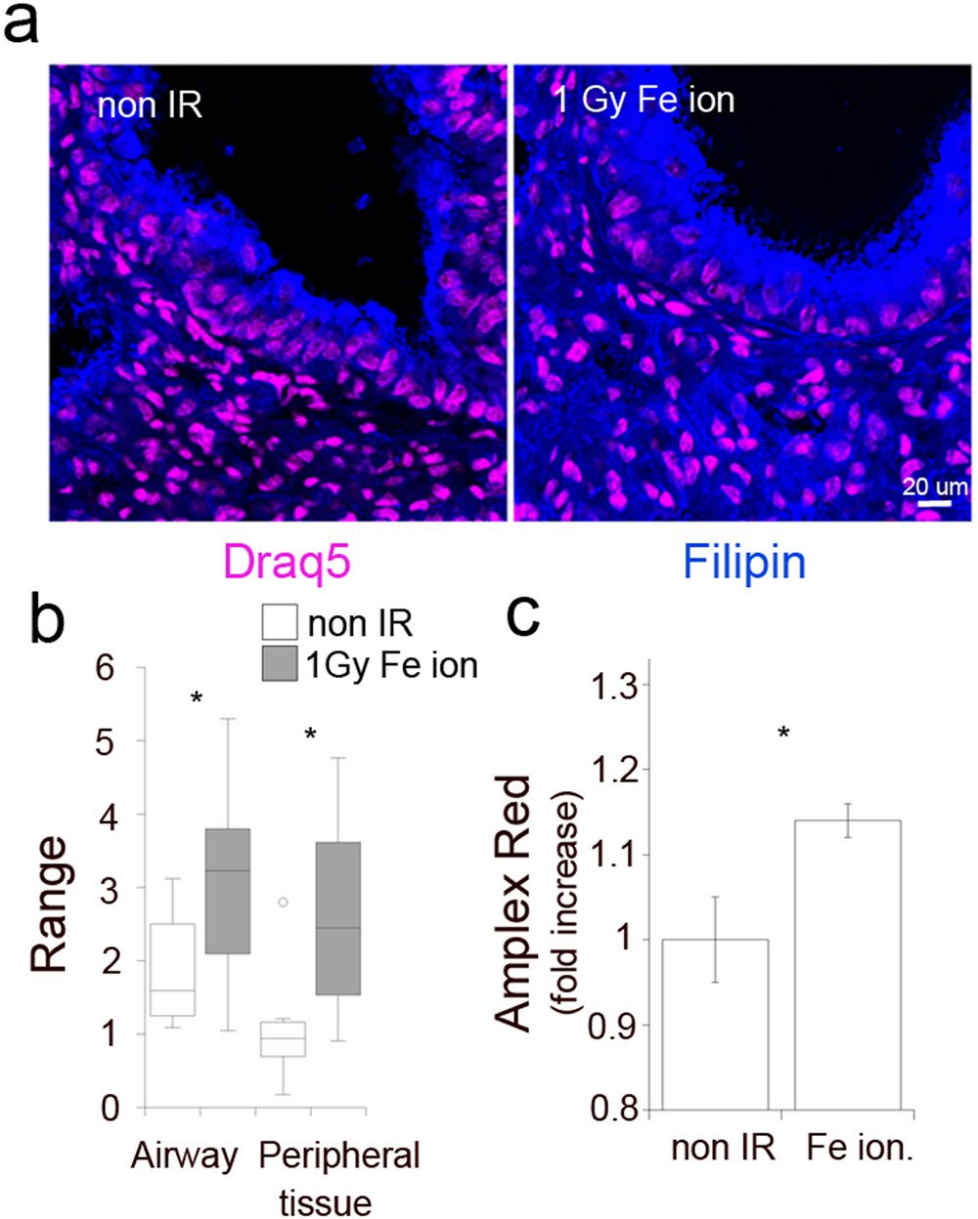
Exposure to Fe ion radiation increases lung tissue associated cholesterol. (**a**) Confocal fluorescence microcopy of lung tissue sections from a non-irradiated or a 1 Gy Fe ion whole body irradiated mouse, stained for cell-associated cholesterol with filipin (blue) and nuclear DNA with Draq5 (purple). Arrows point to the airway bronchial epithelium and respiratory tissue. (**b**) Average fluorescence intensity of filipin staining in the bronchial epithelium (Airway) and the nearby respiratory tissue (Peripheral tissue) was quantified employing Fiji/Image J. The box plots represent measurements in 8 different areas. Paired T-Test, *p < 0.05. (**c**) Total cholesterol content measured with the Amplex Red assay in lysates prepared from frozen lung tissue isolated from mock and 1 Gy Fe ion whole body irradiated mouse. Average of duplicate measurements in two different tissue samples. Paired T-Test, *p < 0.05.

To test whether our *in vitro* model reproduces phenotypes induced in lung tissue, we stained frozen lung tissue sections extracted from whole body irradiated C57BL/6 mouse that was sacrificed at day 7 post-exposure to 1 Gy Fe ion. Increased filipin fluorescence was observed in the epithelial cells lining small airways and in cells located in the adjacent respiratory compartments (Fig. 3a). Quantification of the integrated fluorescence of both regions (Fig. 3b) demonstrates that exposure to Fe ion increases cholesterol levels in both compartments, indicating that this response is not unique to HBEC3-KT cells and may be shared by multiple cell types in the lung. A significant increase in cholesterol levels in lung tissue homogenates was also detected employing an enzymatic assay (Fig. 3c).

**Figure 4:**
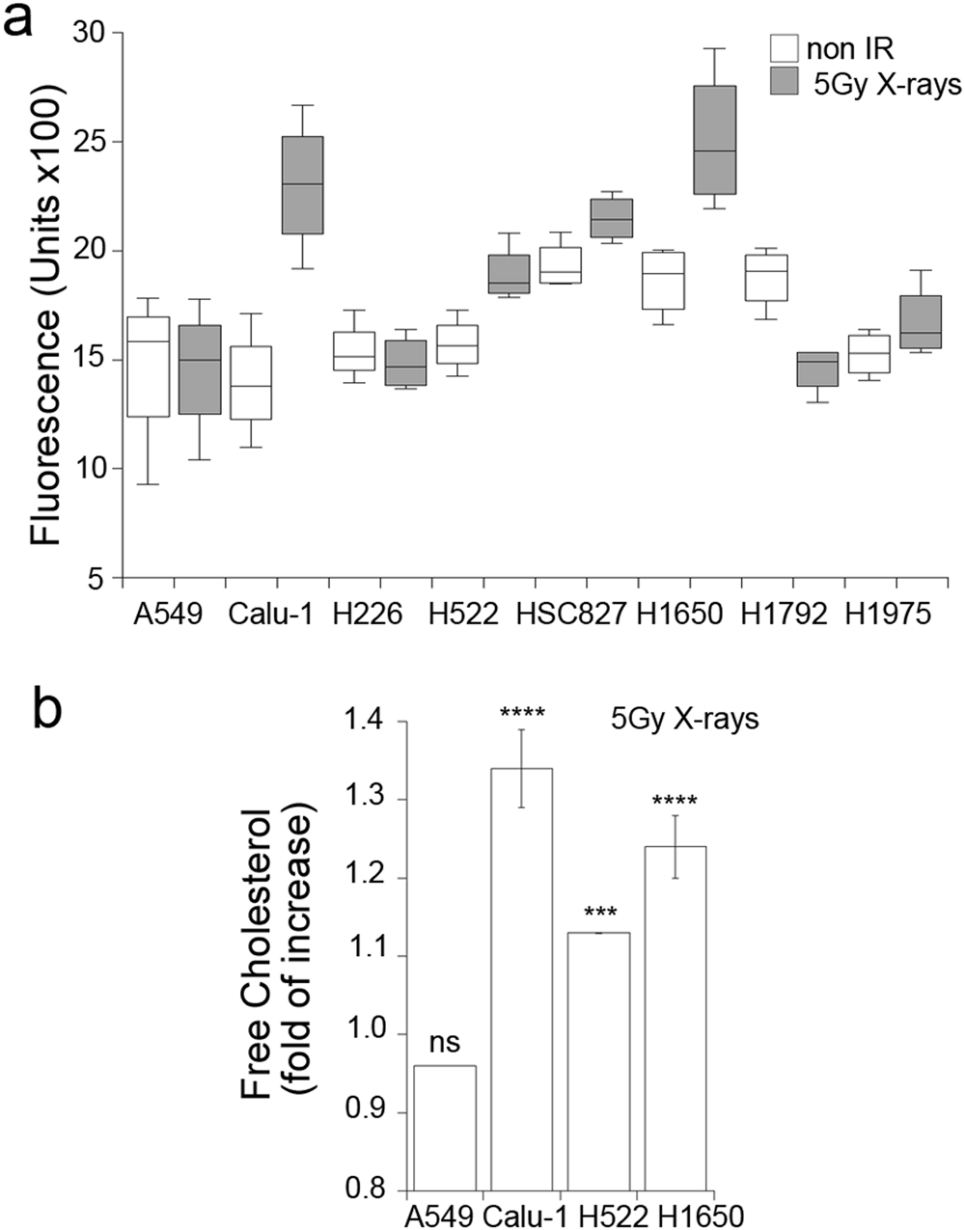
X-rays increases cell-associated cholesterol in a subgroup of non-small cell lung cancer cell lines. (**a**) Average fluorescence intensity of filipin staining in a panel of lung cancer cell lines 48 h following mock or 5 Gy X-rays exposure. (1 of 2 replicate experiments shown). Error bars represent standard deviations of triplicate measurements. (**b**) Total cholesterol content measured with the Amplex Red assay in cell lines that increase cholesterol content following exposure to 5 Gy X-rays Error bars represent standard error of 3 replicate experiments. One-way ANOVA with Dunnett’s post test F = 16, p < 0.005. ***p < 0.005.

Because of the potential toxic effects of cholesterol on cells, we evaluated next whether this response is elicited by low LET radiation in cancer cell lines and modulates radiosensitivity in a dose range relevant to radiation therapy. We measured cholesterol levels in a panel of lung cancer cell lines employing filipin staining in a 96-well plate assay format 48 h following exposure to 5 Gy X-rays, a dose that induced cholesterol accumulation in normal cells. In several of the 8 cell lines tested, X-rays increased cholesterol levels in variable magnitudes, while in two of them (A549 and H226) the levels were not affected (Fig. 4a). Cholesterol induction did not correlate with p53, Ras or EGFR mutation status of the cell lines. Employing the Amplex Red assay, we quantified cholesterol increases in Calu-1, H522 and H1650 which were the cell lines most responsive to radiation demonstrating increases of 10% in H522, 20% in H1650 and 30% in Calu-1 compared to no change in A549 (Fig. 4b).

**Figure 5:**
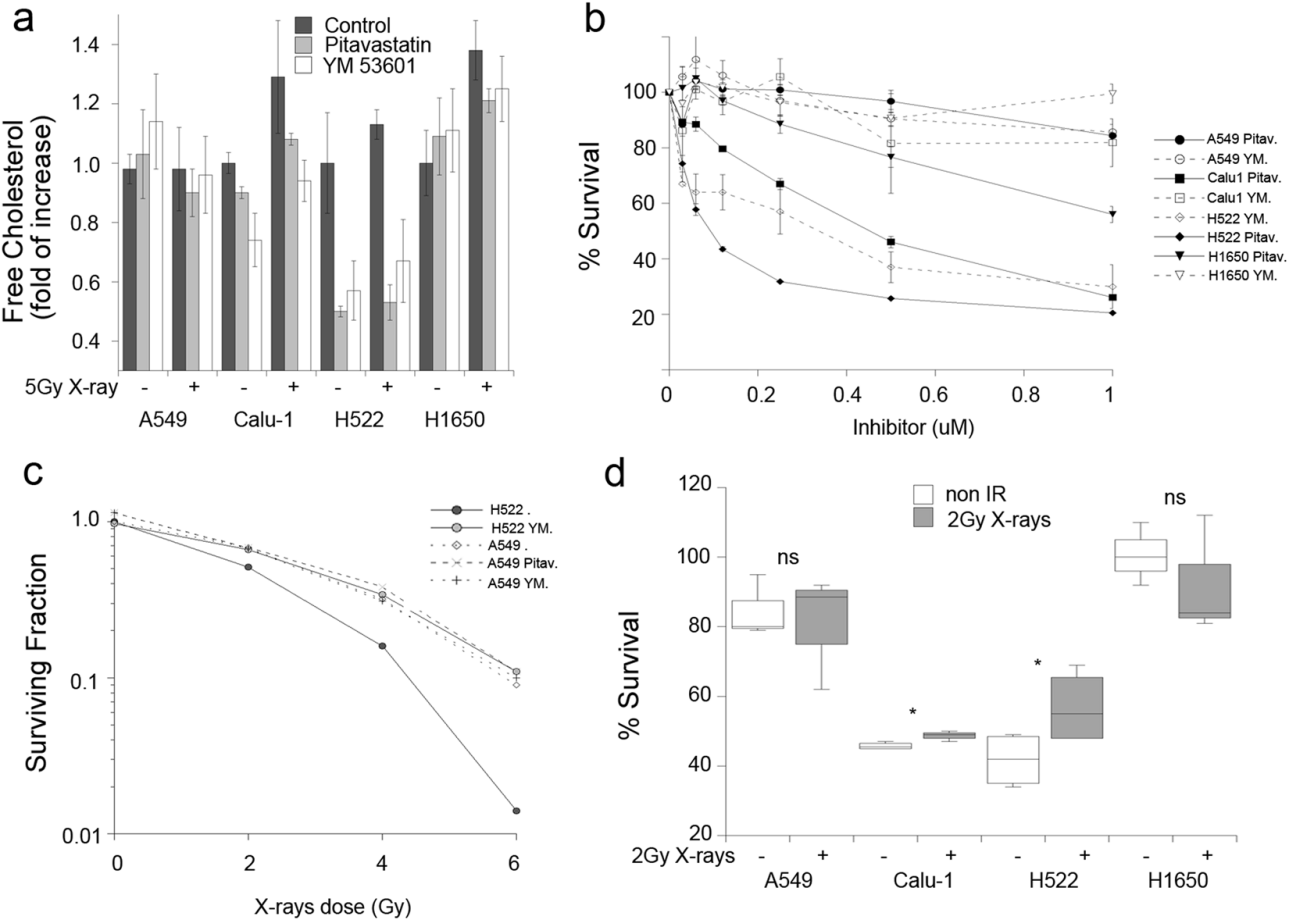
Effect of cholesterol biosynthesis inhibition on cell growth and radiosensitivity. (**a**) Cellular cholesterol levels measured with the Amplex Red assay are reduced by an 18 h treatment prior the assay with vehicle (black bars), 1 μM Pitavastatin (grey bars) or 2 μM YM 53601 (empty bars) in non-irradiated and 5 Gy X-rays irradiated cells. (**b**) MTT survival assay following cholesterol biosynthesis inhibition in non-irradiated cells. In the 3 cell lines were radiation induced cholesterol synthesis, statins reduce cell growth while in H522 both inhibitors reduce cell growth. One of two experiments is shown. Error bars represent standard deviation of triplicate samples. (**c**) Effect of the cholesterol synthesis inhibitor YM 53601 on A549 (dotted line) or H522 (full line) cell survival measured by the clonogenic assay following exposure to X-rays. 1 of 2 experiments is shown. Error bars represent standard deviation of triplicate samples. (**d**) Cells were irradiated with 2 Gy X-rays and within the hour treated with 1.25 μM (A549, Calu1 and H1650 or 0.3 μM (H522) Pitavastatin. Cell growth was measured by MTT reduction after 72 h and compared to non-irradiated cells. The average of 3–4 experiments is shown. Paired t-test. NS = not significative, *p < 0.05.

To test whether cholesterol synthesis modulates cellular radiosensitivity we measured the biological effects of pathway inhibition targeting 3-Hydroxy-3-Methyl glutaryl-CoA Reductase with water- and lipid-soluble statins and Squalene synthase with YM 5603 to examine the effects of inhibiting the synthesis of metabolites downstream of mevalonate. An 18 h treatment with these inhibitors reduced the radiation-induced upregulation of cholesterol levels (Fig. 5a), supporting our previous finding that exposure to radiation induces *de novo* synthesis of cholesterol. The inhibitors reduced basal levels of cholesterol in two cell lines, Calu-1 and H522, indicating that *de novo* synthesis contributes to the steady-state pool of cholesterol in these cell lines. When we assessed the effect of the inhibitors on cell growth in the absence of radiation by MTT reduction, only Calu-1 and H522 were sensitive to Pitavastatin (IC50 of 1.35 μM +/− 0.34 SE and 0.2 μM +/− 0.1 SE respectively), and only H522 was sensitive to the Squalene Synthase inhibitor YM 5603 with an IC50 of 4.25 μM +/− 0.72 SE (Fig. 5b). These results highlight the heterogeneity of lung cancer cell lines reliance on different sources for cholesterol and dependency on the cholesterol biosynthetic pathway for survival.

To test whether cholesterol synthesis modulates cell survival following exposure to radiation, we first examined the effects of pathway inhibition on colony survival following X-ray exposure in two cell lines able to grow in sparse conditions required to measure clonogenic growth. Inhibition of Squalene Synthase increased the clonogenic survival of H522 cells but not of A549 (Fig. 5c), suggesting a contribution of cholesterol accumulation to radiation toxicity. We tested the effects of modulating cholesterol synthesis with statins on cell growth at an earlier time point employing the MTT assay to monitor cell number following exposure to a radiotherapy relevant dose of 2 Gy, which is the approximate equivalent of one fraction. Treatment of the cells with a dose close to the IC50 of Pitavastatin increased the survival of Calu-1 and H522, but not of A549 or H1650 (Fig. 5d).

## Discussion

An unbiased analysis of the proteome of cells that have been exposed to radiation revealed that induction of the cholesterol biosynthesis pathway is a major response persisting at day 7 in irradiated lung epithelial cell lines and in lung tissue. We show for the first time that radiation up-regulates the expression of four enzymes in the cholesterol biosynthesis pathway with functional consequences as demonstrated by increased cellular content of cholesterol following exposure to low and high LET radiation. A radiation dose as low as 0.5 Gy of high LET was sufficient to induce this response, which persisted at day 7-post exposure *in vitro* and *in vivo*. Fe ion were more effective increasing cholesterol, with a relative biological effectiveness (RBE) of 5 estimated from cholesterol levels in exposed cells measured with Amplex Red in Fig. 2, panels a and b. A dose response experiment to determine enzyme expression induced by low LET radiation (Fig. 1, panel d), suggest that this is a threshold type response.

The mechanism for cholesterol accumulation, which we demonstrate by two different methods, is by inducing cholesterol biosynthesis and is supported by increases in biosynthetic enzyme expression levels and by sensitivity of the phenotype to inhibitors targeting four different enzymes of the cholesterol biosynthetic pathway. The enzymes detected in the proteome are genes transcriptionally upregulated by SREBP and downregulated by cholesterol as part of a feedback loop that maintains cholesterol homeostasis^14,15^. Therefore, it is intriguing that we can detect increased cholesterol levels at day seven after irradiation. While we did not perform an extensive time course analysis to determine when this phenotype emerges and how long it persists, our findings suggest the activity of an additional modification that alters the normal negative feedback mechanisms contributing to the expression of this phenotype long after the initial damage to DNA has been repaired. Known mechanisms that disrupt feedback regulation of cholesterol are inflammation, metabolic syndrome and mTOR activation^16^ and as part of lipid reprogramming associated with cancer progression^17–19^. Very few studies have followed up gene and protein expression data with actual cholesterol measurements. Our mass spectrometry results indicated increased FADS1 and FADS2 expression, the rate limiting enzymes in the synthesis of polyunsaturated fatty acids, which have been shown to affect multiple health outcomes and the response to other DNA damaging agents as well^12,20^. Other concurrent phenotypes induced by radiation could influence radiosensitivity, including previously shown production of IL1α and several other inflammatory cytokines^8^ as well as oxidative stress and genomic instability. Thus in this work we provide evidence for a novel condition (exposure to radiation) that alters cholesterol homeostasis by inducing biosynthesis and, potentially, loss of feedback regulation.

While the radiation-induced increases in cholesterol levels are apparently modest (between 10 and 30%), the magnitude of the increases detected is well within the range of changes that affect protein and cellular function. The majority of the cell’s cholesterol (60 to 90%) is concentrated in the plasma membrane^21^, where it regulates protein function indirectly by affecting the biophysical properties of the lipid bilayer or directly by binding to specific motifs in target proteins. This cholesterol pool is regulated around a set point with a narrow range^21,22^ and is sensitive to small changes (10–20%) in concentration^23^. The activity of proteins sensitive to cholesterol, which include ABC sterol transporters, the Na,K-ATPase, nicotinic acetylcholine receptors, three potassium ion channels, the ligand-gated ion channel GABA receptor, and the NMDA glutamate-gated ion channel, respond to modest changes of cholesterol in their local membrane environment. Furthermore, every G protein-coupled receptor has binding sites for cholesterol or their function is affected by sterol (reviewed in^24^). Therefore, we can safely speculate that the changes we detected ranging between 10 and 30% in cholesterol levels are within a physiological range and are expected to modify the function of susceptible proteins.

We detected increased cholesterol levels induced by radiation in multiple systems, including non-tumorigenic cells, cancer cell lines and in lung tissue from a whole body irradiated mouse, where a generalized increase in cholesterol levels is observed in multiple compartments of lung tissue. This finding suggests that a similar response could occur in other post-mitotic tissues that are high risk for radiation exposures such as the cardiovascular and central nervous systems. The effects of low dose radiation on cardiovascular disease are still controversial, however increased incidence of cardiovascular disease is observed in the atomic bomb survivors cohort^25^ and cardiovascular disease is a known side effect of radiation therapy^26–28^. Another potentially susceptible tissue is the central nervous system, where cholesterol cannot be transported across the blood-brain barrier, and therefore is synthesized *de novo*^29,30^. Oxysterols have also been used as biomarkers in neurodegenerative diseases^31^.

Previous studies have explored the effects of cholesterol biosynthesis and mevalonate levels on radiosensitivity at the moment of exposure, with conflicting outcomes probably due to variations in the accumulated product or cell-specific context. Increased toxicity of mevalonate has been attributed to the accumulation of oxysterols^32^, while radioprotective effects have been attributed to the regulation of DNA repair^33^ mediated by activation of small GTPases^34^ or to still undefined protective pathways^35^. Our findings support the conclusion that accumulated cholesterol contributes to radiation toxicity in cell lines that are dependent on this pathway for cell growth and are relevant for fractionated radiotherapy as accumulation occurs after 2 days of exposure. While our findings support statin use as a therapeutic strategy in a selective fashion because they affected cell growth of some of the cell lines in the absence of radiation, they also suggest that statins may not be effective in combination with radiation as they reduced radiosensitivity.

For normal tissue radioprotection, statins are safe, well tolerated and have broad pleiotropic effects, including immunomodulatory, anti-inflammatory and neuroprotective effects^36–38^. Simvastatin and Lovastatin have been shown to reduce radiation induced inflammatory and pro-fibrotic markers without affecting DNA repair in normal tissues^39,40^. Thus, statin use poses many advantages as a mitigation agent by, in addition to reducing cholesterol synthesis, targeting other radiation-induced alterations in addition to reducing cholesterol synthesis^41^.

## Methods

### Irradiation experiments

Experiments utilizing mice were carried out in accordance with the American Association for Laboratory Animal Science policies and were approved by the Emory University and Brookhaven National Laboratory Institutional Animal Care Use Committee (IACUC). Both institutions are AAACLA accredited. Animals were housed in filter-top cages and were kept under standard laboratory conditions of temperature, pressure and humidity with a 12:12 h light–dark schedule. Mice were provided purified sterilized water ad libitum and fed a standard, commercial laboratory animal diet milled by Purina Lab Diets. A six week old C57BL/6 female mouse (Jackson Laboratory, Bar Harbor, ME) was whole-body irradiated with a dose of 600 MeV/nucleon Iron (=175 keV/μm), 50 cGy dose rate at the NASA Space Radiation Laboratory (NSRL) Brookhaven National Laboratory. The NSRL dosimetry group calibrated the ion energy. Data was collected from triplicate irradiations in experiments over 3 different campaigns. Low-LET irradiation was performed at the Winship Cancer Institute of Emory University using a X-RAD 320 × -ray machine (320 kV, 10 mA Precision X-Ray, North Branford, CT).

### Cell culture

The human bronchial epithelial cell line (HBEC3-KT) was a gift from Dr. Story (UT Southwestern) and was authenticated by karyotyping. Cells were cultured in Keratinocyte serum free media (Invitrogen) supplemented with antibiotics, Epidermal growth Factor and Bovine Pituitary extract. For Fe ion irradiations, cells were plated in triplicate T25 flasks at 30% confluence. Each flask was sub-cultured 1:3 on day 4 and plated for the assays on day 6 post-exposure. A549, H1650, H1975, H522, H226, H1792 and HCC827 cell lines were maintained in RPMI-1640 media supplemented with 10% FBS, and were obtained from the Winship Cancer Institute (Atlanta, GA, USA).

### Label-free quantitative global proteomic analysis

Three T25 flasks for each experimental condition were independently mock or 0.5 Gy Fe irradiated, passaged at day 4 and the cells were harvested at day 7 and stored frozen until analysis. Samples were homogenized in 8 M urea buffer and 100 μg of protein homogenates were diluted with 50 mM NH_4_HCO_3_ to a final concentration of less than 2 M urea and proteins were reduced, alkylated and digested with LysC/trypsin as previously described^42^. Desalted peptides (2 μg) were analyzed by nanoliquid chromatography coupled to tandem mass spectrometry (nanoLC-MS/MS) on a Fusion Orbitrap (Thermo Fisher Scientific, San Jose, CA). Elution was performed over a 120 minute gradient at a rate of 300 nl/min with buffer B ranging from 1% to 65% (buffer A: 0.1% formic acid in water, buffer B: 0.1% formic in acetonitrile). The machine was set to collect at top speed for cycles of 3 seconds where ms1 scans were collected in the Orbitrap at 120,000 resolution with an automatic gain control (AGC) of 200,000, a m/z range of 400–1600, and a max injection time of 50 ms. Precursor ions were fragmented at 32% normalized collision energy (NCE) in the Higher-energy C-trap dissociation (HCD) cell and collected in the ion trap with an AGC of 10000 and a max injection time of 35 ms. Other parameters include S-lens RF of 60%, dynamic exclusion of 20 s, exclusion of +1 and greater than +7 charged species, and quadrupole isolation window of 0.7 Da. The spectra were searched using the Andromeda search engine against a human Uniprot database (downloaded April 2015 – 90270 target sequence). The search parameters include full tryptic enzymatic cleavage, 0.6 Da fragment mass tolerance, dynamic methionine oxidation (+15.994915), N-terminal acetylation (+42.010565), and static cysteine carbamidometyl (+57.021464). Peptide-specific ion current intensities were extracted and compared using MaxQuant (version 1.5.2.8) label-free quantification (LFQ) according to standard procedures as described^43^. Accurate peptide mass and retention time were defined by MaxQuant derived signal intensity for every peptide across LC-MS/MS runs for each sample. For each pair-wise cohort comparison, GenePattern (The Broad Institute) was employed to produce a statistically analyzed list of proteins, each ranked by the degree of change and p-value significance of the abundance change it exhibits between specified cohorts as we have previously shown^44^. From a total of 2706 proteins quantified and identified, 51 proteins varied significantly after filtering for the following criteria established previously: (a) a cut-off of >2 SD for upregulated and 0.5 < for downregulated proteins (outside 95% confidence interval), (b) with a p value < 0.05 and (c) passed a threshold set at 5% for FDR estimated by utilizing the power of technical replicates and a null experimental comparison to quantify false positives under current filtering criteria^44,45^. The list of proteins shown in supplementary material was analyzed employing the functional annotation tools with default settings in the Database for Annotation, Visualization and Integrated Discovery (DAVID). Data is available at Synapse.org, 10.7303/syn18081952, https://www.synapse.org/#!Synapse:syn18081952/files/.

### Western blot analysis

Cell lysates were preparing employing RIPA buffer (50 mM Tris pH = 7.4, 150 mM NaCl, 2 mM EDTA, 0.5% NP40, 0.25% Sodium Deoxycholate) supplemented with Complete protease inhibitor and StopPHOS phosphatase inhibitor (Roche). Hundred micrograms of protein were separated in 10% SDS PAGE and transferred to a PVDF membrane. The membrane was probed with antibodies for HMGCS1 (Abcam 194971) and SQLE (Proteintech 12544-1-AP). Tubulin was detected as a loading control (Sigma, T6074). Bound antibodies were detected with infrared fluorescent secondary antibodies and imaged with a LI-COR Odyssey system. The intensity of the bands was quantified employing Image Studio Software. The individual bands were corrected by loading and then divided by the units detected in non-irradiated (0 Gy) samples to obtain the values shown below each band.

### Cholesterol content measurements

#### Amplex red cholesterol assay

100,000 cells were plated 24 h before lysis in 100 μl Buffer A (10 mM HEPES, pH 7.4, 150 mM NaCl, 1 mM EGTA, and 0.1 mM MgCl_2_) containing 0.5% Triton X100 supplemented with antiproteases (Complete, Roche). Lysates from frozen lung tissue were prepared in Buffer A 0.5% Triton X100 with 10 strokes of a Dounce homogenizer, sonicated 3 times for 10 seconds at 6 watts and centrifuged at maximum speed in a refrigerated tabletop centrifuge. Protein concentration was equalized in all samples at 2.5 μg/μl for tissue lysates and at 1 μg/μl for cell lysates. Seventy five microliters of lysate were extracted with 300 μl of a 3:1 mixture of chloroform: isopropanol. A hundred and fifty microliters of the organic phase were recovered and the solvents evaporated at room temperature in a speed-vac. The lipids were resuspended in 50 μl of reaction buffer and cholesterol was measured as recommended by the Amplex® Red Cholesterol Assay kit (Invitrogen). This is an enzyme coupled fluorimetric assay that detects both free cholesterol and cholesteryl esters.

#### Filipin staining

Cells plated on glass coverslips, in 96 well black wall plates or frozen tissue sections were fixed with 4% Paraformaldehyde in PBS. After 4 washes with PBS, aldehyde groups were quenched with 10 mM Glycine in PBS for 10 minutes. Filipin from *streptomyces filipensis* (Sigma) was added at a dilution of 0.05 mg/ml in PBS containing 10% fetal bovine serum and incubated for 2 h at room temperature in the dark. After four washes with PBS, nuclear DNA was counterstained with 5 μM Draq5 (eBioscience) for fluorescence microscopy assays or SybrGreen (Invitrogen) for plate assays. Cell associated filipin (350 nm ex/450 nm em) and SybrGreen (480 nm ex/580 nm em) fluorescence was measured in a Molecular Dynamics Plate reader. Cells and tissue sections were imaged in a Olympus epifluorescence inverted microscope and a Zeiss LSM510META confocal microscope (Thornwood, NY, USA) using a 20 × Plan-Apo objective (NA = 0.75). Images were processed using contrast/brightness enhancement only. Filipin fluorescence in the images was quantified employing Fiji (Image J) recording the integrated fluorescence intensity for filipin and for Draq5 fluorescence, a far-red cell-permeable DNA intercalating probe to correct for cell density. In tissue images, two ROI of defined area (4 × 4 arbitrary units) were placed on the bronchial epithelium lining small airways or on the adjacent respiratory portion in four different images. In cultured cells, the cell-associated pixel intensity of fillipin fluorescence was recorded for a total of 50–70 cells per sample in replicate irradiations.

### Tissue preparation and staining

Lung tissue was collected from mice euthanized at day 7 post-irradiation, and snap frozen in liquid nitrogen to preserve molecular integrity of the cells and for laser scanning microscopy imaging. The tissues were embedded in OCT Compound (Tissue-Tek) and cut frozen into five micrometer thick sections. Frozen sections were thawed in 3% PFA and stained with filipin as described above.

### Cell growth and survival assays

To measure cell growth, cells were plated in 96 well plates and irradiated and/or treated with inhibitors 18 h after plating. Cell growth was measured by quantification of reduced MTT and IC50 were calculated with GraphPad Prism employing non-linear regression of an equation for the logarithm of the inhibitor concentration vs. % survival with a variable slope and a top constrain of less or equal to 100. Cell survival to radiation was measured by the colony survival assay 12 days following exposure. Inhibitors were added within one hour of exposure to radiation and washed after 3 days.

### Statistical analysis

The statistical test applied in each case is stated in the figure legend. Excel was used for paired two-tailed student’s t-test analysis assuming equal variance of the samples. Graph Pad Software (San Diego, CA, USA) was used for repeated measures analysis of variance employing a Bonferroni (repeated measures) or Dunnett’s (one way) post-hoc test.

## Supplementary information

Supplementary Information

## Data Availability

Mass spectrometry raw data is available at Synapse.org, 10.7303/syn18081952, https://www.synapse.org/#!Synapse:syn18081952/files/.
