# Supplementary material for: Ionizing Radiation induction of cholesterol biosynthesis in Lung tissue
Source: Sci Rep. 2019 Aug 29;9:12546. doi: 10.1038/s41598-019-48972-x (PMC6715797; doi:10.1038/s41598-019-48972-x)

Supplementary Information:

# "Ionizing Radiation induction of cholesterol biosynthesis in Lung tissue"

Erica Werner, Andrew Alter, Qiudong Deng, Eric B. Dammer, Ya Wang, David S. Yu, Duc M. Duong, Nicholas T. Seyfried and Paul W. Doetsch

# Supplementary Table 1: List of proteins regulated by Fe ion exposure.

| Gene symbol | Name of the protein | Protein.IDs | f01 | f02 | f03 | n01 | n02 | n03 | log.p | Pvalue | threshold | Differen | fold change(N/ | Fold (F/N |
|---|---|---|---|---|---|---|---|---|---|---|---|---|---|---|
| ALDH3A1 | Aldehyde dehydrogenase 3 fam | tr\|C9JMC5\|C9JMC5_HUMA | 7.49601 | 7.8445 | 7.978 | 8.4173 | 8.714 | 8.347 | -1.773 | 0.0169 | TRUE | -0.72 | 5.250043 | 0.1904 |
| HSPB1 | Heat shock protein beta 1 | sp\|P04792\|HSPB1_HUMAN | 9.16436 | 9.1934 | 9.147 | 9.3701 | 9.721 | 9.559 | -1.694 | 0.0202 | TRUE | -0.38 | 2.408609 | 0.4151 |
| BCAM | Basal cell adhesion molecule | tr\|A0A087WXM8\|A0A087W | 7.47551 | 7.4963 | 7.547 | 7.6879 | 8.117 | 7.82 | -1.34 | 0.0457 | TRUE | -0.37 | 2.337496 | 0.4278 |
| CA2 | Carbonic anhydrase 2 | sp\|P00918\|CAH2_HUMAN; | 7.66075 | 7.6376 | 7.462 | 7.8712 | 8.095 | 7.805 | -1.451 | 0.0354 | TRUE | -0.34 | 2.173935 | 0.4 |
| PTK7 | Protein Tyrosine Kinase 7 | sp\|Q13308-3\|PTK7_HUMA | 7.74636 | 7.9971 | 7.86 | 8.0956 | 8.303 | 8.128 | -1.471 | 0.0338 | TRUE | -0.31 | 2.031725 | 0.4921 |
| SPRR1B | Cornifin B | sp\|P22528\|SPR1B_HUMAN | 7.97578 | 7.8686 | 7.942 | 8.1469 | 8.381 | 8.157 | -1.65 | 0.0224 | TRUE | -0.3 | 1.993225 | 0.501 |
| AKR1C2 | Aldo-keto reductase family 1 m | sp\|P52895\|AK1C2_HUMAN | 7.82009 | 7.863 | 7.891 | 8.0114 | 8.241 | 8.155 | -1.778 | 0.0167 | TRUE | -0.28 | 1.895976 | 0.5274 |
| PCK2 | Phosphoenolpyruvate carboxyk | sp\|Q16822\|PCKGM_HUMA | 8.33044 | 8.3008 | 8.362 | 8.5664 | 8.637 | 8.579 | -3.144 | 0.0007 | TRUE | -0.26 | 1.833137 | 0.5455 |
| CTSD | Cathepsin D | sp\|P07339\|CATD_HUMAN; | 9.12262 | 9.1886 | 9.149 | 9.2976 | 9.56 | 9.376 | -1.491 | 0.0323 | TRUE | -0.26 | 1.810984 | 0.5521 |
| HBB | Hemoglobin beta | CON__P02070;CON__Q3SX | 7.8013 | 7.6399 | 7.777 | 7.9511 | 7.936 | 8.065 | -1.713 | 0.0194 | TRUE | -0.24 | 1.756484 | 0.5693 |
| HIST1H1C | Histone H1C | sp\|P16403\|H12_HUMAN;s | 9.6761 | 9.852 | 9.73 | 9.9863 | 9.975 | 9.967 | -1.887 | 0.013 | TRUE | -0.22 | 1.672775 | 0.5978 |
| DNAJC2 | DnaJ homolog subfamily C mem | sp\|Q99543-2\|DNJC2_HUM | 7.53481 | 7.3096 | 7.434 | 7.6012 | 7.644 | 7.669 | -1.447 | 0.0358 | TRUE | -0.21 | 1.628764 | 0.6139 |
| FAHD1 | Acylpyruvase | sp\|Q6P587\|FAHD1_HUMA | 7.77815 | 7.9304 | 7.848 | 8.0288 | 8.137 | 8.013 | -1.617 | 0.0242 | TRUE | -0.21 | 1.612476 | 0.6201 |
| ECH1 | Delta(3,5)-Delta(2,4)-dienoyl-C | sp\|Q13011\|ECH1_HUMAN; | 8.04727 | 8.1538 | 8.088 | 8.2642 | 8.364 | 8.283 | -2.049 | 0.0089 | TRUE | -0.21 | 1.612273 | 0.6202 |
| H1FX | Histone 1x | sp\|Q92522\|H1X_HUMAN | 8.34009 | 8.3921 | 8.368 | 8.6281 | 8.484 | 8.6 | -1.921 | 0.012 | TRUE | -0.2 | 1.59852 | 0.6255 |
| MUT | Methylmalonyl-CoA mutase | sp\|P22033\|MUTA_HUMAN | 7.286 | 7.1965 | 7.152 | 7.4097 | 7.438 | 7.366 | -1.908 | 0.0124 | TRUE | -0.19 | 1.560255 | 0.6409 |
| SDCBP | Syntenin-1 | sp\|O00560\|SDCB1_HUMAN | 8.16473 | 8.1505 | 8.154 | 7.9816 | 7.97 | 7.991 | -4.675 | 2.11E-05 | TRUE | 0.175 | 0.667743 | 1.4975 |
| TTI1 | TELO2-interacting protein 1 hor | sp\|O43156\|TTI1_HUMAN | 7.75163 | 7.6808 | 7.637 | 7.4246 | 7.569 | 7.538 | -1.503 | 0.0314 | TRUE | 0.179 | 0.661801 | 1.5110 |
| SCARB1 | Scavenger receptor class B men | sp\|Q8WTV0-4\|SCRB1_HUM | 7.77851 | 7.8861 | 7.795 | 7.569 | 7.62 | 7.728 | -1.461 | 0.0346 | TRUE | 0.181 | 0.659439 | 1.5164 |
| RBMS2 | RNA-binding motif, single-stran | sp\|Q15434\|RBMS2_HUMA | 7.89264 | 7.7022 | 7.791 | 7.5856 | 7.602 | 7.646 | -1.473 | 0.0337 | TRUE | 0.184 | 0.654854 | 1.5270 |
| TTC37 | Tetratricopeptide repeat protei | sp\|Q6PGP7\|TTC37_HUMAN | 7.91382 | 7.7741 | 7.869 | 7.6294 | 7.736 | 7.628 | -1.582 | 0.0262 | TRUE | 0.188 | 0.649082 | 1.5406 |
| LAMC2 | Laminin subunit gamma-2 | sp\|Q13753\|LAMC2_HUMA | 9.23567 | 9.2228 | 9.175 | 9.0962 | 8.992 | 8.981 | -1.994 | 0.0101 | TRUE | 0.188 | 0.648302 | 1.5424 |
| WAPL | Wings apart-like protein homol | sp\|Q7Z5K2\|WAPL_HUMAN | 7.0271 | 6.9376 | 7.043 | 6.8891 | 6.733 | 6.806 | -1.591 | 0.0257 | TRUE | 0.193 | 0.640663 | 1.5608 |
| LPXN | Leupaxin | sp\|O60711\|LPXN_HUMAN; | 8.45487 | 8.5606 | 8.528 | 8.2634 | 8.33 | 8.35 | -2.101 | 0.0079 | TRUE | 0.2 | 0.630612 | 1.5857 |
| CSK | Tyrosine-protein kinase CSK | sp\|P41240\|CSK_HUMAN;tr | 7.96223 | 7.9202 | 7.991 | 7.71 | 7.645 | 7.853 | -1.571 | 0.0268 | TRUE | 0.221 | 0.600552 | 1.6651 |
| SPATS2 | Spermatogenesis-associated se | sp\|Q86XZ4\|SPAS2_HUMAN | 8.74995 | 8.7372 | 8.699 | 8.4128 | 8.601 | 8.499 | -1.785 | 0.0164 | TRUE | 0.225 | 0.596101 | 1.6775 |
| CDKN2AIPNL | CDKN2AIP N-terminal-like prote | sp\|Q96HQ2\|C2AIL_HUMAN | 8.22037 | 8.1923 | 8.019 | 7.8805 | 7.828 | 8.015 | -1.311 | 0.0488 | TRUE | 0.236 | 0.581137 | 1.7207 |
| CYP51A1 | Lanosterol 14-alpha demethyla | sp\|Q16850\|CP51A_HUMAN | 8.6308 | 8.5143 | 8.586 | 8.3878 | 8.39 | 8.245 | -1.8 | 0.0159 | TRUE | 0.236 | 0.580568 | 1.7224 |
| FSTL1 | Follistatin-related protein 1 | sp\|Q12841-2\|FSTL1_HUMA | 6.7959 | 6.819 | 6.776 | 6.6571 | 6.524 | 6.499 | -2.023 | 0.0095 | TRUE | 0.237 | 0.579648 | 1.7251 |
| ATP6V1H | V-type proton ATPase subunit H | sp\|Q9UI12-2\|VATH_HUMA | 7.97132 | 8.132 | 8.028 | 7.8374 | 7.884 | 7.685 | -1.471 | 0.0338 | TRUE | 0.242 | 0.573063 | 1.7450 |
| HiGD1A | HIG1 domain family member 1A | tr\|C9JAW5\|C9JAW5_HUMA | 8.03587 | 8.2226 | 8.062 | 7.7755 | 7.859 | 7.955 | -1.452 | 0.0353 | TRUE | 0.244 | 0.570472 | 1.7529 |
| AAAS | Aladin | tr\|F8VZ44\|F8VZ44_HUMAN | 7.88056 | 7.7601 | 7.679 | 7.6426 | 7.521 | 7.413 | -1.31 | 0.0489 | TRUE | 0.248 | 0.565317 | 1.7689 |
| THOC3 | THO complex subunit 3 | sp\|Q96J01\|THOC3_HUMAN | 8.1398 | 8.0464 | 8.121 | 7.8539 | 7.98 | 7.723 | -1.462 | 0.0345 | TRUE | 0.25 | 0.562218 | 1.7786 |
| FADD | FAS-associated death domain p | sp\|Q13158\|FADD_HUMAN | 8.41752 | 8.2342 | 8.383 | 8.0198 | 8.061 | 8.192 | -1.532 | 0.0294 | TRUE | 0.254 | 0.557322 | 1.794 |
| UCK2 | Uridine-cytidine kinase 2 | sp\|Q9BZX2\|UCK2_HUMAN; | 7.94037 | 7.9337 | 7.854 | 7.798 | 7.57 | 7.555 | -1.492 | 0.0322 | TRUE | 0.268 | 0.539327 | 1.8541 |
| CDK4 | Cyclin-dependent kinase 4 | sp\|P11802\|CDK4_HUMAN; | 9.8375 | 9.8062 | 9.83 | 9.5575 | 9.455 | 9.534 | -3.17 | 0.0007 | TRUE | 0.309 | 0.490678 | 2.03 |
| MSMO1 | Methylsterol monooxygenase 1 | sp\|Q15800-2\|MSMO1_HU | 7.90859 | 8.0245 | 7.968 | 7.6981 | 7.628 | 7.638 | -2.84 | 0.0014 | TRUE | 0.312 | 0.487105 | 2.0529 |
| SQLE | Squalene monooxygenase | tr\|E7EVQ6\|E7EVQ6_HUMA | 8.02901 | 7.8967 | 7.89 | 7.6894 | 7.723 | 7.457 | -1.527 | 0.0297 | TRUE | 0.315 | 0.483857 | 2.0667 |
| FN1 | Fibronectin | sp\|P02751-6\|FINC_HUMAN | 7.99319 | 7.964 | 7.953 | 7.6453 | 7.642 | 7.663 | -4.722 | 1.90E-05 | TRUE | 0.32 | 0.478235 | 2.0910 |
| DUT | Deoxyuridine 5'-triphosphate n | sp\|P33316-2\|DUT_HUMAN | 8.434 | 8.4649 | 8.371 | 8.2349 | 7.892 | 8.167 | -1.397 | 0.0401 | TRUE | 0.325 | 0.473068 | 2.1138 |
| RFTN1 | Raft-linking protein, isoform CR | tr\|G3XAJ6\|G3XAJ6_HUMAN | 7.79599 | 7.8211 | 7.772 | 7.4566 | 7.321 | 7.593 | -1.883 | 0.0131 | TRUE | 0.34 | 0.457588 | 2.1853 |
| IPO11 | Importin-11 | sp\|Q9UI26\|IPO11_HUMAN | 8.2006 | 8.2281 | 8.143 | 7.8818 | 7.802 | 7.808 | -3.247 | 0.0006 | TRUE | 0.36 | 0.436384 | 2.2915 |
| FADS2 | Fatty acid desaturase 2 | sp\|O95864\|FADS2_HUMAN | 8.61977 | 8.5036 | 8.553 | 8.2661 | 8.096 | 8.231 | -2.371 | 0.0043 | TRUE | 0.361 | 0.435635 | 2.295 |
| TRIP12 | E3 ubiquitin-protein ligase TRIP | sp\|Q14669-4\|TRIPC_HUMA | 7.76426 | 7.9081 | 7.634 | 7.4494 | 7.231 | 7.449 | -1.662 | 0.0218 | TRUE | 0.392 | 0.405216 | 2.4678 |
| THBS1 | Thrombospondin-1 | sp\|P07996\|TSP1_HUMAN;s | 9.15713 | 9.0691 | 9.105 | 8.8049 | 8.602 | 8.689 | -2.52 | 0.003 | TRUE | 0.412 | 0.387663 | 2.5795 |
| HMGCS1 | Hydroxymethylglutaryl-CoA syn | sp\|Q01581\|HMCS1_HUMA | 9.4041 | 9.3766 | 9.384 | 9.0986 | 8.804 | 9.021 | -2.022 | 0.0095 | TRUE | 0.413 | 0.386047 | 2.5903 |
| FADS1 | Fatty acid desaturase 1 | sp\|O60427\|FADS1_HUMAN | 7.83135 | 7.5282 | 7.768 | 7.108 | 7.193 | 7.46 | -1.5 | 0.0316 | TRUE | 0.456 | 0.350233 | 2.8552 |
| IL1A | Interleukin-1 alpha | sp\|P01583\|IL1A_HUMAN | 8.08982 | 7.9666 | 7.976 | 7.6892 | 7.517 | 7.452 | -2.318 | 0.0048 | TRUE | 0.458 | 0.348204 | 2.8718 |
| F3 | Tissue factor | sp\|P13726\|TF_HUMAN;sp\| | 8.33194 | 8.3969 | 8.203 | 8.0594 | 7.521 | 7.819 | -1.433 | 0.0369 | TRUE | 0.511 | 0.308524 | 3.2412 |
| ENAH | Protein enabled homolog | sp\|Q8N8S7\|ENAH_HUMAN | 7.30695 | 7.5495 | 7.766 | 6.8331 | 6.435 | 7 | -1.67 | 0.0214 | TRUE | 0.785 | 0.164152 | 6.091 |

Western Blots without cropping:

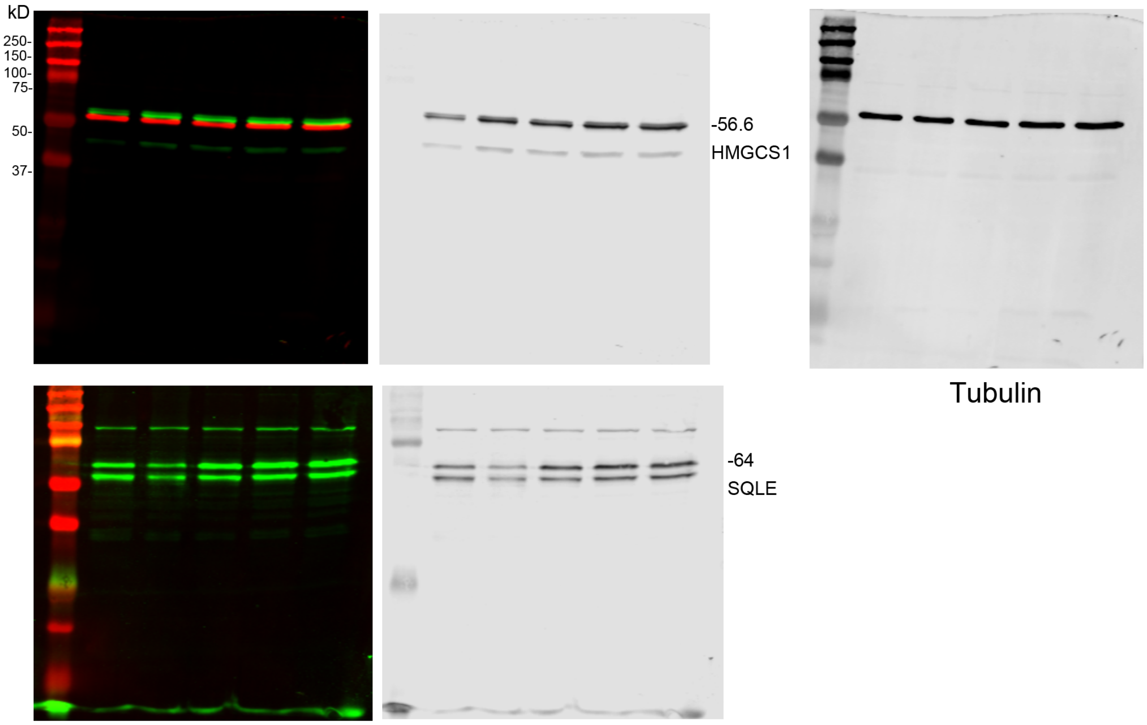

Supplement: Supplementary file 1 — Supplementary Information [file 41598_2019_48972_MOESM1_ESM.pdf]
